# Simulation and experimental study on control strategy of zero-speed fin stabilizer based on disturbance and compensation

**DOI:** 10.1371/journal.pone.0204446

**Published:** 2018-10-01

**Authors:** Lihua Liang, Peng Zhao, Songtao Zhang, Ming Ji, Jiguang Song, Jia Yuan

**Affiliations:** College of Automation, Harbin Engineering University, Harbin, Heilongjiang, China; University of Birmingham, UNITED KINGDOM

## Abstract

Zero-speed fin stabilizer is applied to reduce the roll motion of ships at zero speed. This paper aims to explore the control strategy of zero-speed fin stabilizer through a composite method of theoretical analysis, simulations and tank tests. The hydrodynamic force model is established using analytical approach and a simplified model is obtained by fitting the CFD simulation data. The control strategy of zero-speed fin stabilizer is obtained based on disturbance and compensation by analyzing the phase matching relationship between the wave disturbance, the roll motion of the ship, the movement of the fin and the fin-induced hydrodynamic force. Simulations and water tank tests are performed to verify the effectiveness and feasibility of the obtained control strategies. The results of simulations and tank tests show that the obtained control strategies of zero-speed fin stabilizer based on disturbance and compensation are effective and practical. The proposed method provides theoretical and experimental support for engineering application, and can also be a reference for the controller design of zero-speed fin stabilizers.

## 1 Introduction

A ship in a seaway moves in six degrees of freedom (6-DOF) under the interference of sea winds, waves and currents. Compared with the other five degrees of freedom, the roll motion of the ship, which is mainly caused by the waves, has the largest impact on the safety of the ship. Large roll motion may lead to cargo damage, on-board operation interruption and even ship capsizing [1, 2]. With the development of ocean engineering, more and more on-board operations need to be performed at low and zero speed, such as working boat’s lowering and hoisting, helicopter’s taking-off and landing [3]. Moreover, ships experience larger roll motion at zero speed as the roll damping decreases with ship speed [4]. Therefore, it is necessary to reduce the roll motion of the ship at zero speed.

Bilge keel, anti-rolling tank, moving weight and gyrostabiliser are the commonly used roll reduction devices at zero speed. Bilge keel is a passive anti-rolling device mounted at the bilge on both sides of the ship. It reduces ship roll motion by increasing ship’s roll damping, but it also increases the sailing resistance [5]. The anti-rolling tank reduces ship’s roll motion through water’s reciprocating movement. It takes up valuable cabin space to achieve a satisfactory roll reduction effect [6]. Moving weights generate restoring torque by controlling the movement of the weights, which requires large power consumption [7]. Gyrostabiliser produces stabilization torque through the high-speed rotation of the flywheel. However, its anti-rolling capacity is limited by its size and it is usually installed on small yachts [8].

Fin stabilizers are the most effective active anti-rolling devices. Their anti-rolling effect at high sailing speed can be up to 90% in theory [9]. But their performance at low and zero speed is poor. The most feasible option for ship roll reduction with satisfactory anti-rolling effect at whole speed range is the integrated roll reduction system of anti-rolling tank and fin stabilizer [10]. The integrated system uses the anti-rolling tank to realize the roll reduction at low and zero speed, and uses both the anti-rolling tank and fin stabilizer to reduce the roll motion at medium/high speed. However, the cost to design, install and maintain two separate devices is huge. It also takes up valuable cabin space, and increases ship’s displacement and sailing resistance. Therefore, the best way to resolve the above problem is to improve the conventional fin stabilizer to make it have the anti-rolling ability at whole speed, and the improved one is called the zero-speed fin stabilizer [11–14]. It adopts the normal work mode at medium/high speed and switches to the zero-speed mode at zero/low speed [15]. The biggest advantage of this design is its compatibility with the conventional fin stabilizer system. The cost will not increase too much as we only need to design and maintain one system. Zero-speed fin stabilizer was firstly proposed in 1998 by the researchers and engineers of Amels and MARIN in Netherlands [16]. The intended purpose was to improve the comfort of motor yachts at anchor. A pair of fin stabilizers were used to generate the anti-rolling force to reduce ship’s roll motion at zero speed using the principle of paddle [16, 17]. The world’s first zero-speed fin stabilizer system was installed on a 71-meter-long yacht named Boadicea by Naiad, MARIN and Amels in 1999. The new system significantly reduced the roll motion of the ship at zero speed and its anti-rolling effect under speed condition is also satisfactory [18, 19]. Another zero-speed anti-rolling system based on the Weis-Fogh mechanism was proposed by the Institute of Ship Stabilization and Control Research of Harbin Engineering University in 2005 [20, 21]. The proposed scheme could produce large restoring force to stabilize ship’s roll motion at zero speed, but it cannot be practically applied due to its complex structure. Therefore, the longitudinal flapping zero-speed fin stabilizer proposed by MARIN and Quantum Control is considered in this paper.

Dallinga [16] proposed a bang-bang controller to control the zero-speed fin stabilizers to reduce ship’s roll motion at zero speed. Jin and Zhang [22] designed a fuzzy controller with minimum energy consumption based on an improved genetic algorithm to reduce the roll motion at zero speed. Jin and Wang [23] proposed a variant constraint model predictive controller to control the fins, however, the lift constraint and special working method limit its roll reduction effect. Song and Liang [24] designed a zero-speed fin stabilizer controller based on radial basis function and general regression neural network. Su and Gao [25] designed a fuzzy sliding mode controller to stabilize the roll motion. The above papers directly give the controller design for zero-speed fin stabilizers according to its hydrodynamic force characteristics. In this paper, the control strategy is obtained based on disturbance and compensation by analyzing the phase matching of the wave disturbance, the roll motion of the ship, the movement of the fin and the fin-induced hydrodynamic force. An 84-meter-long fishery administration ship was selected as the target ship, and all the simulations and tank tests were carried out on the ship. The structure of this paper is as follows. Section 2 establishes the hydrodynamic force model of the zero-speed fin stabilizer. Section 3 analyzes the control strategy of zero speed fin stabilizer based on disturbance and compensation. Section 4 establishes the simulation model of zero-speed fin stabilizer roll reduction system and gives the controller design. Section 5 verifies the applicability of the obtained control strategies through simulations and water tank tests. Finally, the conclusion is given.

## 2 Hydrodynamic modeling for zero-speed fin stabilizer

### 2.1 Ship configuration

An 84-meter-long fishery administration ship was selected as the research object. Two pairs of fin stabilizers are designed to satisfy its roll damping requirement at zero speed. The principle parameters of the ship and the designed zero-speed fin stabilizer are shown in Tables 1 and 2, respectively. The scaled model of the fishery administration ship was also built, with the scale ratio of 1:25, to conduct the water tank test. The parameters of the scaled models of the ship and the fin are also listed in Tables 1 and 2, respectively.

**Table 1:** Main parameters of the ship.

Description	Symbol	Prototype	Model
Length between perpendiculars (m)	*L*	84	3.36
Beam over all (m)	*B*	10	0.4
Draft (m)	*d*	3.2	0.128
Displacement (t)	*D*	1300	0.0832
Transverse metacentric height (m)	*h*	1.1	0.04
Roll period (s)	*T*_*φ*_	8.5	1.7
Dimensionless decay coefficient	*n*_*u*_	0.12	0.12

**Table 2:** Main parameters of the fins.

Description	Symbol	Prototype	Model
Fin area (m^2^)	*A*	3.92	0.006272
Aspect ratio	Λ	0.5	0.5
Chord (m)	*c*	2.8	0.112
Span (m)	*s*	1.4	0.056
Maximum fin angle (°)	*α*_*max*_	±60	±60
Maximum fin rate (°/s)	${\dot{\alpha }}_{max}$	45	225
Roll arm (m)	*l*_*f*_	5.7	0.228

### 2.2 Hydrodynamic modeling

**Fig 1:**
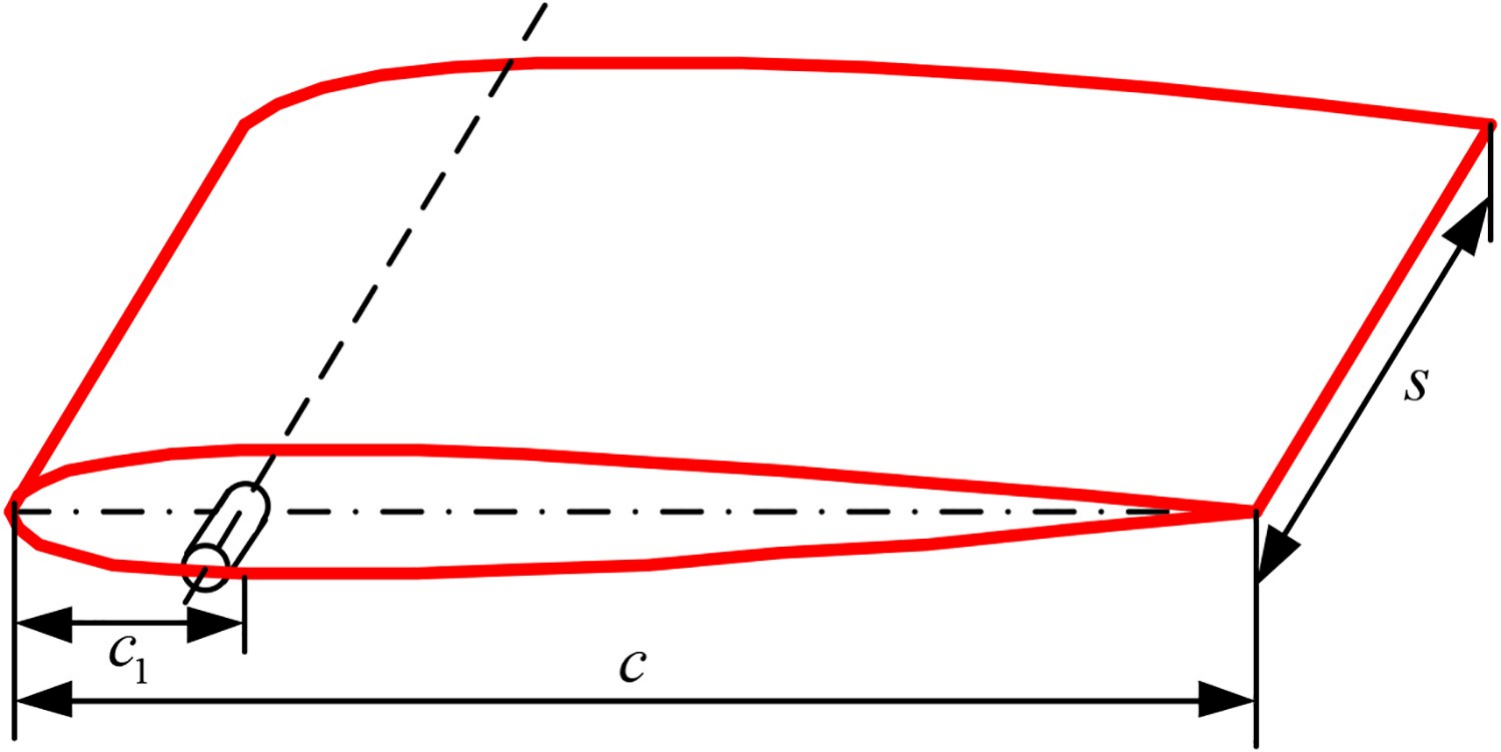
Prototype model of zero-speed fin stabilizer.

The fin rotating about its shaft in a non-ideal flow field is subjected to the pressure drag, friction drag, vortex drag and added inertia force [11, 16]. Compared with the pressure drag, vortex drag and added inertia force, the frictional stress is much smaller and generally can be ignored [26]. Therefore, the hydrodynamic force on the active flapping fin is a composite force of the pressure drag, vortex drag and added inertia force. In this paper, the NACA0015 fin, as shown in Fig 1, is selected as a prototype to model the hydrodynamic force, where *c* is the chord, *c*_1_ is the distance between the leading edge and the fin shaft, and *s* is the span.

#### 2.2.1 Pressure drag force

**Fig 2:**
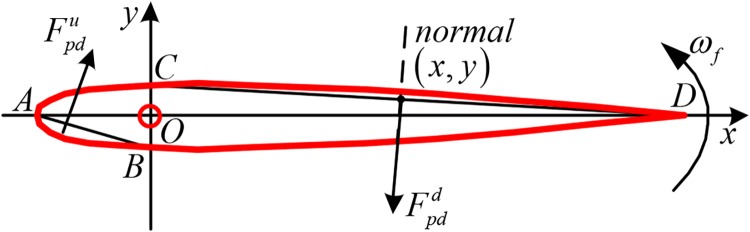
Diagram of the pressure drag force.

The pressure drag force is closely related to the shape and movement of the fin[27]. The Cartesian coordinate system is defined as shown in Fig 2. To simplify the analysis, the arc segment $\hat{CD}$ is replaced by the straight line $\bar{CD}$.

When the fin rotates around its shaft at angular rate *ω*_*f*_, the instantaneous pressure drag of any element (*x*, *y*) on the rear side of the fin shaft can be expressed as:

$$\begin{array}{c} dF_{pd}^{d}=\frac{1}{2}C_{D}\rho v^{2}sdl \end{array}$$ (1)

Where *C*_*D*_ is the drag coefficient, *ρ* is the fluid density, $v=\omega_{f}\sqrt{x^{2}+y^{2}}$ is the instantaneous normal velocity of the element (*x*,*y*) and *l* is the length of the straight line $\bar{CD}$.

Integrating Eq (1), we get:

$$\begin{array}{c} F_{pd}^{d}=\frac{1}{2}C_{D}\rho s\omega_{f}^{2}\sqrt{1+\frac{{\bar{t}}^{2}}{{\left(c-c_{1}\right)}^{2}}}(\frac{{\left(c-c_{1}\right)}^{3}}{3}+\frac{{\bar{t}}^{2}(c-c_{1})}{3}) \end{array}$$ (2)

Where $\bar{t}$ is the thickness of the fin.

Compared with the chord *c* and span *s*, the thickness $\bar{t}$ is much smaller and generally can be ignored. Therefore, Eq (2) can be simplified as:

$$\begin{array}{c} F_{pd}^{d}=\frac{1}{6}C_{D}\rho s\omega_{f}^{2}{\left(c-c_{1}\right)}^{3} \end{array}$$ (3)

Similarly, the pressure drag on the front side of the fin shaft is obtained as:

$$\begin{array}{c} F_{pd}^{u}=\frac{1}{6}C_{D}\rho s\omega_{f}^{2}{c_{1}}^{3} \end{array}$$ (4)

Therefore, the total pressure drag is:

$$\begin{array}{c} F_{pd}=F_{pd}^{d}-F_{pd}^{u}=\frac{1}{6}C_{D}\rho s\omega_{f}^{2}[{\left(c-c_{1}\right)}^{3}-{c_{1}}^{3}] \end{array}$$ (5)

#### 2.2.2 Added inertia force

The added inertia force arises from the inertia of the fluid. When flapping the fin up and downwards, a certain mass of fluid is accelerated or decelerated. However, their inertia always tries to keep them in the initial motion state and consequently gives a force called the added inertia force to the fin [20]. Let *T* be the kinetic energy of the fluid and *J* the added moment of inertia of the fin, then *T* can be calculated as:

$$\begin{array}{c} T=\frac{1}{2}J\omega_{f}^{2} \end{array}$$ (6)

Let *L* = *T* − *P* be the Lagrange function, where *P* is the potential energy of the fin surface. As the distance between the fin shaft and the centre of gravity of the fin is small, the effect of potential energy *P* on the fin can be ignored. Therefore, the Lagrange function can be expressed as:

$$\begin{array}{c} L=T=\frac{1}{2}J\omega_{f}^{2} \end{array}$$ (7)

According to the second Lagrange equation, we get:

$$\begin{array}{c} \frac{d}{dt}[\frac{\partial L}{\partial \omega_{f}}]=M \end{array}$$ (8)

Where *M* is the fin’s driving torque.

Substituting Eq (7) into Eq (8), we get:

$$\begin{array}{c} J{\dot{\omega }}_{f}=M \end{array}$$ (9)

Let *k*_*c*_(*c*/2 − *c*_1_) be the distance between the acting point of the added inertia force and the fin shaft, then the added inertia force *F*_*ad*_ can be calculated as:

$$\begin{array}{c} F_{ad}=\frac{Je}{k_{c}(c/2-c_{1})}{\dot{\omega }}_{f} \end{array}$$ (10)

The added moment of inertia *J* can be calculated as [28]:

$$\begin{array}{c} J=\pi \rho {\left(c/2\right)}^{2}{\left(c/2-c_{1}\right)}^{2} \end{array}$$ (11)

Substituting Eq (11) into Eq (10), we get:

$$\begin{array}{c} F_{ad}=\frac{1}{8}k_{a}\pi \rho ec^{2}(c-2c_{1}){\dot{\omega }}_{f} \end{array}$$ (12)

Where *k*_*a*_=1/*k*_*c*_ can be approximated as a constant related to the fin angular acceleration.

#### 2.2.3 Vortex drag force

**Fig 3:**
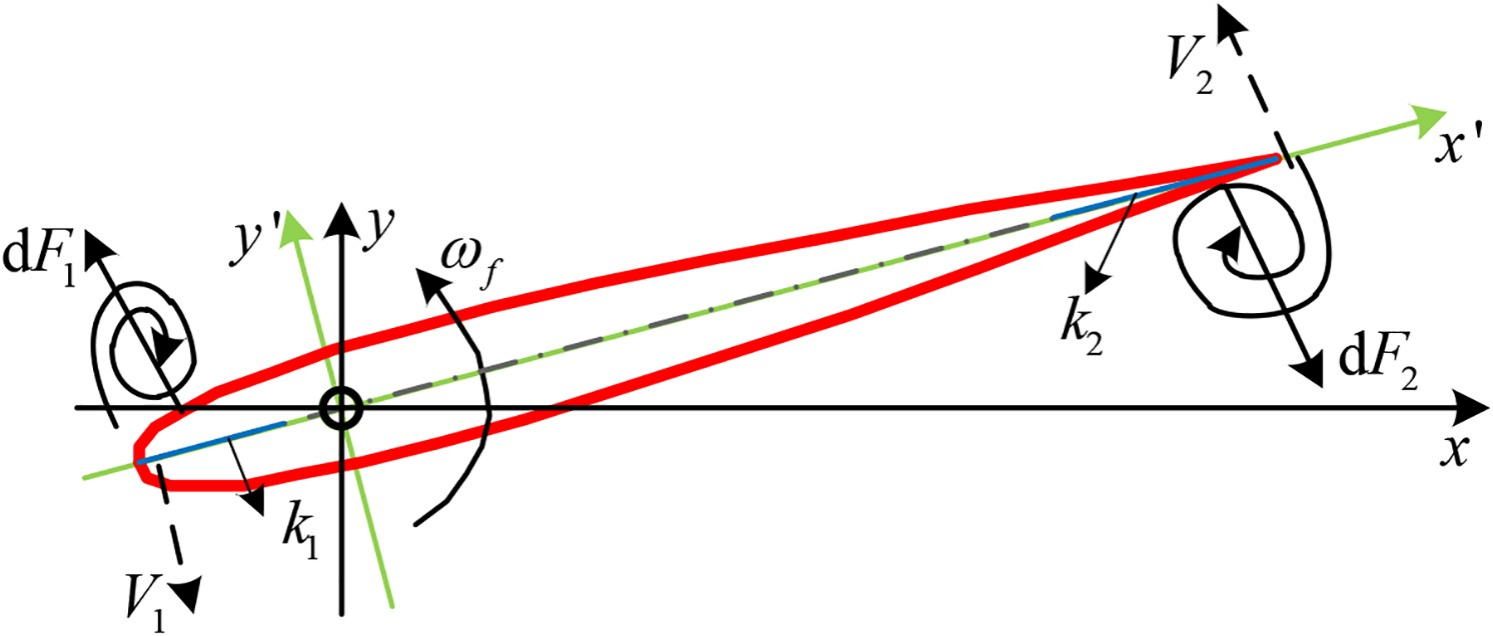
Diagram of the vortex drag force.

The effect of vortex cannot be ignored when the fin rotates in the unsteady flow. Both the leading-edge vortex and the trailing-edge vortex result in the pressure difference on both sides of the fin. The vortex in the unsteady flow increases the movement resistance of the fin and the effect is equivalent to increasing the pressure drag[20, 28]. As shown in Fig 3, *V*_1_ and *V*_2_ are the speeds of the leading and trailing edges, respectively. The outer flow of the leading-edge vortex will fill the gap caused by the movement of the fin at the same speed. Therefore, the induction velocity caused by the leading-edge vortex can be approximated to *V*_1_. In the same way, the induction velocity caused by the trailing-edge vortex can be approximated to *V*_2_.

As shown in Fig 3, the vortex only exists on the side opposite to the fin’s movement. The pressure difference in the front side of the fin shaft can be calculated using Bernoulli theorem as:

$$\begin{array}{c} p_{1}=\frac{1}{2}\rho V_{1}^{2} \end{array}$$ (13)

Let (*x*′, *y*′) be the coordinate of any element on the front side of the fin shaft in the rectangular coordinate *x*′*oy*′. Thus, the linear velocity of the element can be calculated as:

$$\begin{array}{c} {V_{1}}^{\prime }=x^{\prime }\omega_{f} \end{array}$$ (14)

The force generated on the element caused by the leading-edge vortex can be obtained as:

$$\begin{array}{c} dF_{1}=\frac{1}{2}\rho V_{1}^{{}^{\prime }2}sdx^{\prime } \end{array}$$ (15)

The leading-edge vortex is only formed within a certain range of the leading edge. Let *k*_1_ be the length of this limited range, as shown in Fig 3, then the vortex drag generated on the leading edge can be expressed as:

$$\begin{array}{c} F_{1}=\int_{-c_{1}}^{-k_{1}}\frac{1}{2}\rho V_{1}^{{}^{\prime }2}sdx^{\prime } \end{array}$$ (16)

Finishing the integral with Eq (14), we get:

$$\begin{array}{c} F_{1}=\frac{1}{6}\rho s\omega_{f}^{2}(c_{1}^{3}-k_{1}^{3}) \end{array}$$ (17)

Similarly, the vortex drag *F*_2_ generated on the trailing edge can be obtained as:

$$\begin{array}{c} F_{2}=\frac{1}{6}\rho s\omega_{f}s^{2}k_{2}[3{\left(c-c_{1}\right)}^{2}-3k_{2}(c-c_{1})+k_{2}^{2}] \end{array}$$ (18)

Therefore, the total vortex drag force is:

$$\begin{array}{c} F_{vd}=F_{2}-F_{1}=\frac{1}{6}\rho s\omega_{f}^{2}[3k_{2}{\left(c-c_{1}\right)}^{2}-3k_{2}^{2}(c-c_{1})+k_{2}^{3}-(c_{1}^{3}-k_{1}^{3})] \end{array}$$ (19)

It should be noted that the distance between the leading edge and the shaft of the zero-speed fin stabilizer is small and the leading-edge vortex can hardly appears [20]. Therefore, *k*_1_ can be approximated to zero and the vortex drag force *F*_*vd*_ can be simplified as:

$$\begin{array}{c} F_{vd}=\frac{1}{6}\rho s\omega_{f}^{2}[3k_{2}{\left(c-c_{1}\right)}^{2}-3k_{2}^{2}(c-c_{1})+(k_{2}^{3}-c_{1}^{3})] \end{array}$$ (20)

#### 2.2.4 Total hydrodynamic force

The total hydrodynamic force generated on the fin can be expressed as:

$$\begin{array}{c} F=F_{pd}+F_{ad}+F_{vd} \end{array}$$ (21)

Considering their directions, *ω*^2^ in the above equations should be replaced by *ω*|*ω*|. Therefore, the total hydrodynamic force generated on the fin can be obtained as:

$$\begin{array}{c} \begin{array}{c} F=\frac{1}{6}C_{D}\rho s\omega_{f}|\omega_{f}|[{\left(c-c_{1}\right)}^{3}-{c_{1}}^{3}]+\frac{1}{8}k_{a}\pi \rho sc^{2}(c-2c_{1}){\dot{\omega }}_{f}+\\ \;\;\;\;\;\;\;\;\;\frac{1}{6}\rho s\omega_{f}|\omega_{f}|[3k_{2}{\left(c-c_{1}\right)}^{2}-3k_{2}^{2}(c-c_{1})+(k_{2}^{3}-c_{1}^{3})] \end{array} \end{array}$$ (22)

It can be seen from Eq (22) that both the pressure drag and the vortex drag are proportional to the square of the fin angular velocity, while the added inertia force is proportional to the fin angular acceleration. Therefore, Eq (22) can be simplified as:

$$\begin{array}{c} F=K_{1}\rho \omega_{f}|\omega_{f}|+K_{2}\rho {\dot{\omega }}_{f} \end{array}$$ (23)

Where *K*_1_ and *K*_2_ are the coefficients related to the parameters of the fin.

### 2.3 Hydrodynamic force model fitting

It can be seen from Eq (23) that the hydrodynamic force generated on the zero-speed fin stabilizer mainly depends on the angular velocity and acceleration of the fin, which is much different from the conventional lift-based fin stabilizer. The coefficients *K*_1_ and *K*_2_ can be obtained either by theoretical estimation or by fitting the data from computational fluid dynamics (CFD) simulations or tank experiments [16, 20, 28]. Considering the cost and accuracy, the CFD simulation was adopted to obtain these two parameters.

**Fig 4:**
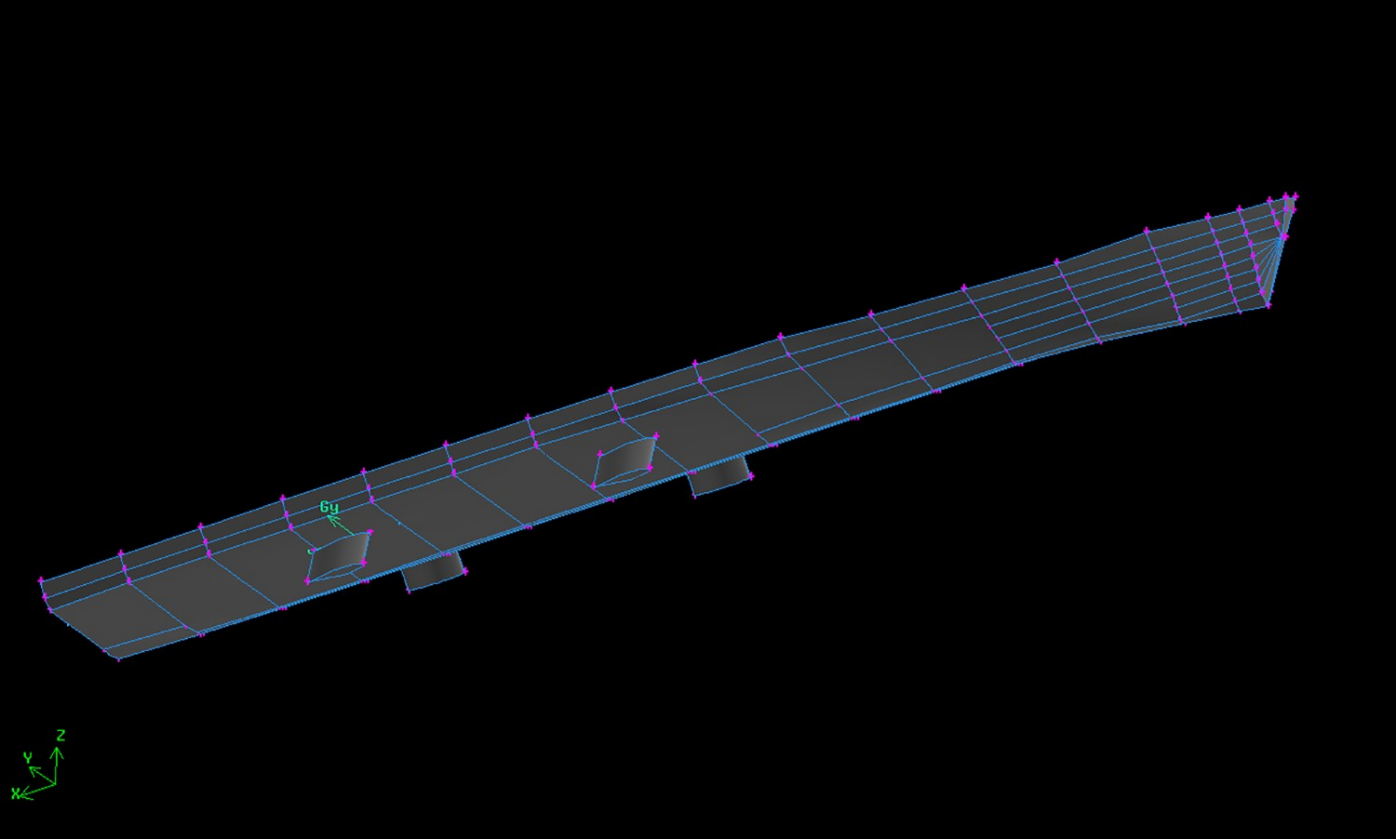
Ship model with fin stabilizers established in GAMBIT.

The commercial CFD software FLUENT was used to simulate the hydrodynamic characteristics of zero-speed fin stabilizer. The model of the fishery ship with two pairs of fin stabilizers, as shown in Fig 4, was built in GAMBIT. The surfaces of the hull and the fins were set as WALL. The SIMPEC algorithm and RNG k-*ε* turbulent model were also adopted. The remeshing method was used to avoid generating negative cells. To improve the precision accuracy of the computation, denser meshes were used where it is closer to the fin. The dynamic mesh technique was used in this simulation. The motion of the fin was defined by the UDF written in C code. The initial velocity of the flow field was set as zero with one standard atmospheric pressure. The fluid was the water with the density of 998.2 kg/m^3^ and the coefficient of the viscosity was 0.001 kg/(m⋅s).

A lot of CFD simulations with different flapping cycles and modes were carried out to fit *K*_1_ and *K*_2_ in Eq (23). Detailed information for the simulations, data fitting and verification process can be found in our previous studies [20, 28–30]. Finally, the parameters *K*_1_ = 20.58 and *K*_2_ = 4.946 were obtained by fitting the data from CFD simulations.

$$\begin{array}{c} F=20.58\rho \omega_{f}|\omega_{f}|+4.946\rho {\dot{\omega }}_{f} \end{array}$$ (24)

## 3 Analysis of disturbance and compensation

According to the Conolly theory [31], the roll motion of the ship equipped with fin stabilizers can be expressed as:

$$\begin{array}{c} (I_{x}+\Delta I_{x})\ddot{\phi }+2N_{u}\dot{\phi }+Dh\phi =-K_{W}-K_{C} \end{array}$$ (25)

Where *ϕ* is the roll angle, *I*_*x*_ is the inertia moment, Δ*I*_*x*_ is the added inertia moment, *N*_*u*_ is the roll damping torque coefficient, *D* is the ship displacement, *h* is the transverse metacentric height, *K*_*W*_ and *K*_*C*_ are the wave disturbance moment and the control torque, respectively.

The ship keeps still when the wave disturbance moment is completely compensated by the fin-induced anti-rolling moment. However, the randomness of the wave disturbance and the limitations of the mechanical system make it difficult to fully compensate for the wave-induced disturbance moment. Therefore, the key problem of roll stabilization is to handle the phase matching problem between the control compensation moment and wave disturbance moment. Assume the ship experiences a sinusoidal roll motion as:

$$\begin{array}{c} \phi =\phi_{a}\cdot \sin(\omega t) \end{array}$$ (26)

Where *ϕ*_*a*_ and *ω* are the amplitude and frequency of the roll motion, respectively.

To reduce the roll motion of the ship, the fin shall move at the same frequency as the roll motion. Therefore, the movement of the fin can be expressed as:

$$\begin{array}{c} \alpha_{f}=\alpha_{a}\cdot \sin(\omega t+\varepsilon ) \end{array}$$ (27)

Where *α*_*f*_ is the fin angle, *α*_*a*_ is the movement amplitude of the fin, *ε* denotes the phase lead of the fin angle with respect to the roll motion.

The angular velocity and acceleration of the fin can be easily obtained:

$$\begin{array}{c} \omega_{f}={\dot{\alpha }}_{f}=\omega \alpha_{a}\cdot \cos(\omega t+\varepsilon ) \end{array}$$ (28)

$$\begin{array}{c} {\dot{\omega }}_{f}={\ddot{\alpha }}_{f}=-\omega^{2}\alpha_{a}\cdot \sin(\omega t+\varepsilon ) \end{array}$$ (29)

Therefore, the hydrodynamic force generated on the fin can be calculated as:

$$\begin{array}{c} F=\rho \omega^{2}\alpha_{a}[K_{1}\alpha_{a}\cos(\omega t+\varepsilon )|\cos(\omega t+\varepsilon )|-K_{2}\sin(\omega t+\varepsilon )] \end{array}$$ (30)

**Fig 5:**
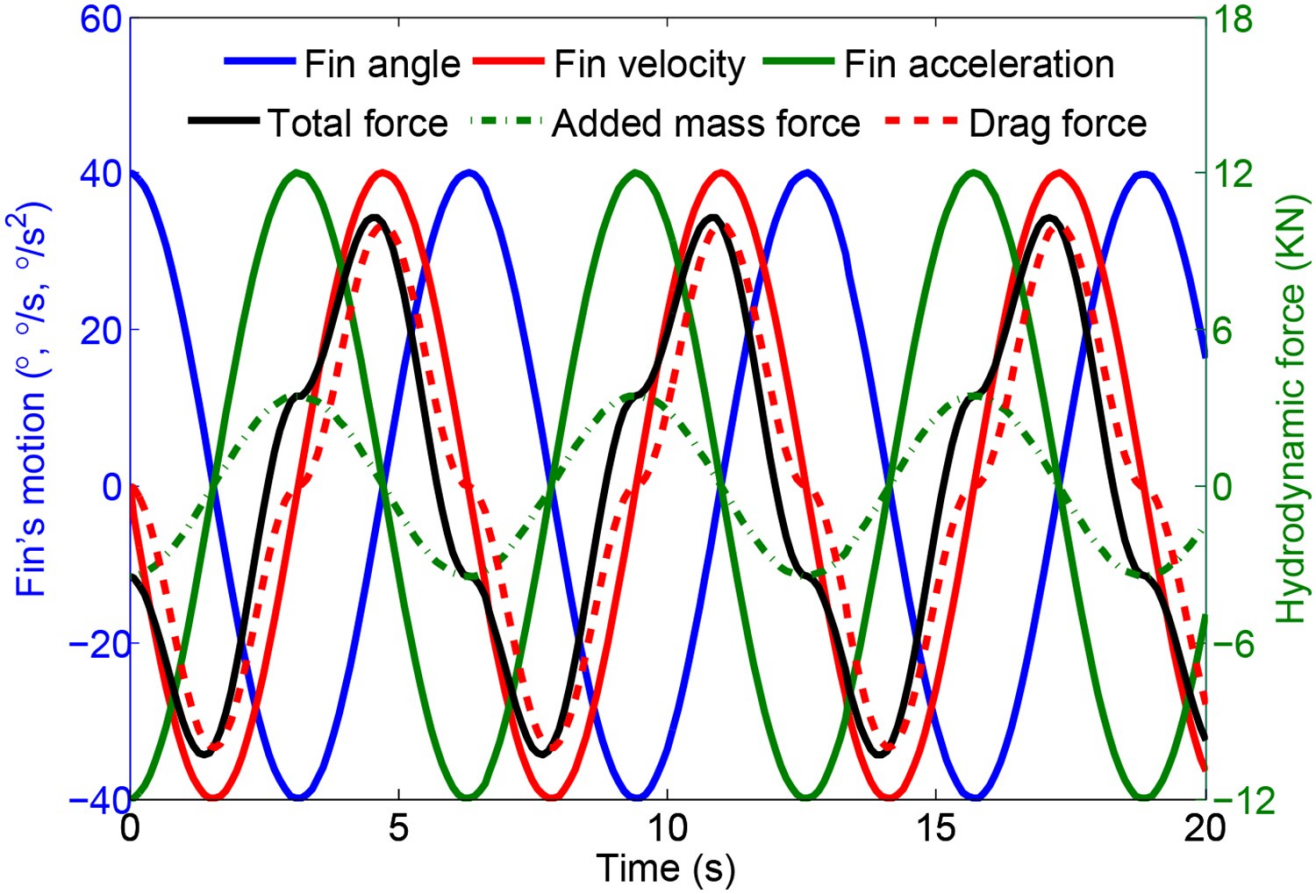
Fin’s movement and hydrodynamic forces generated on it.

Without loss of generality, the parameters are selected as *α*_*a*_ = 40°, *ε* = 30°, *ω* = 1 and *ρ* = 1025 kg/m^3^. The relationship between the movement of the fin and the hydrodynamic forces generated on it are shown in Fig 5. The blue, red and green solid lines represent the fin angle, the fin angular velocity and the fin angular acceleration, respectively. The red dashed line donates the drag force, including the pressure drag and the vortex drag. The green dot-dash line donates the added inertia force. The black solid line describes the total hydrodynamic force.

**Fig 6:**
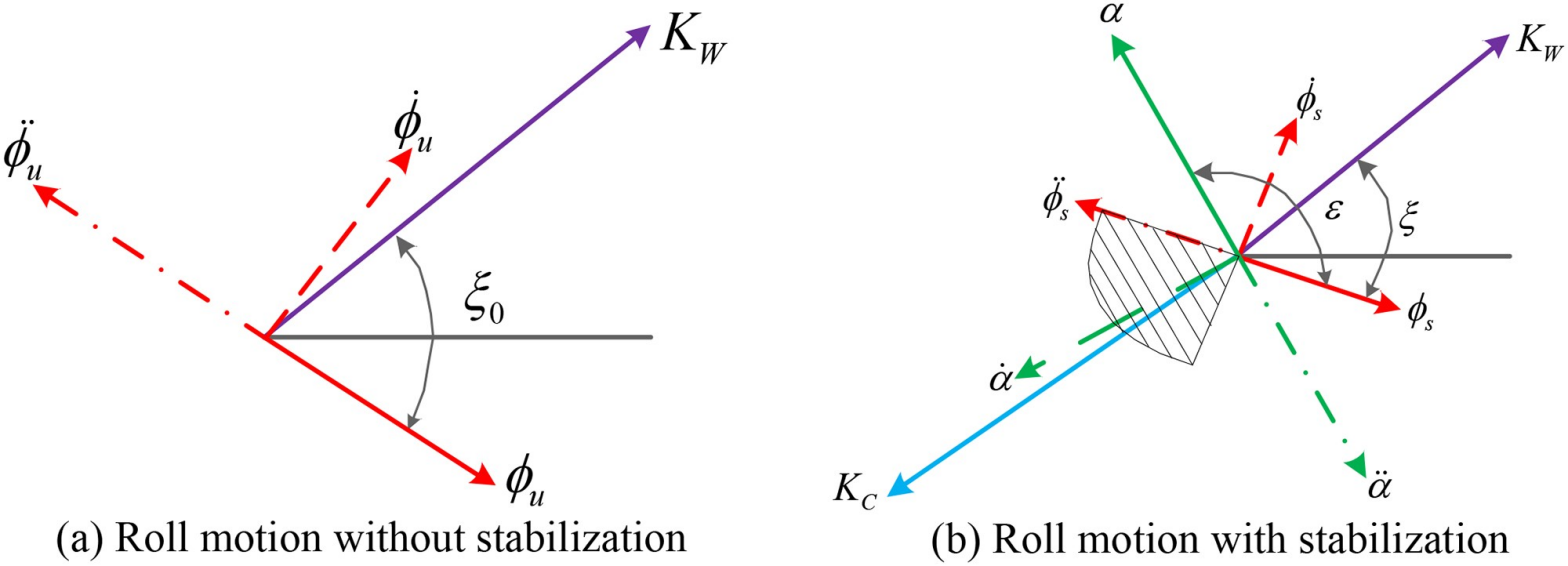
Vector diagram of ship roll motion at zero speed. (a) Roll motion without stabilization. (b) Roll motion with stabilization.

It can be seen from Fig 5 that the phase of the total hydrodynamic force is decided by the fin’s movement. The drag force depends on the fin angular velocity and the added inertia force depends on the fin angular acceleration. The phase of the total hydrodynamic force is just slightly ahead of the phase of the drag force, and the phase of the drag force is the same as the fin angular velocity. Therefore, to reduce the roll motion, the phase relationship between the wave-induced disturbance moment, the roll motion of the ship, the movement of the fin and the fin-induced hydrodynamic force should be as shown in Fig 6. The red solid, dash and dot-dash arrow lines donate the roll angle, roll angular velocity and acceleration, respectively. The green solid, dash and dot-dash arrow lines donate the fin angle, fin angular velocity and acceleration, respectively. The purple solid arrow line represents the wave disturbance moment and the light blue one describes the control compensation moment caused by the fin stabilizers.

The roll motion of the ship without stabilization can be described by the vector diagram shown in the first figure in Fig 6, where *ξ*_0_ donates the phase lag of the roll angle with respect to the wave disturbance moment. In order to reduce the roll motion, the anti-rolling moment induced by the zero-speed fin stabilizers shall compensate for the wave disturbance moment as much as possible. Therefore, the phase of the fin-induced anti-rolling torque shall lead the phase of the wave disturbance moment by *π*. As the phase of the total fin-induced anti-rolling torque is only slightly ahead of the phase of the fin angular velocity, therefore, the vectors of the fin angle, angular velocity and angular acceleration can be obtained, as shown in the second figure in Fig 6. Thus, the phase relationship between the wave disturbance moment, the roll motion of the ship, the movement of the fin and the fin-induced anti-rolling moment is achieved. According to the hydrodynamic characteristics of the zero-speed fin stabilizer and the consideration for engineering implementation, the angular velocity of the fin stabilizer is chosen as the manipulated variable to control the roll motion of the ship at zero speed. The manipulated variable in the control of the conventional fin stabilizers is the fin angle, as the anti-rolling force generated on the conventional fin stabilizer is proportional to the fin angle. The required fin angle of the conventional fin stabilizer is computed based on the roll angle and roll angular velocity feedback signals. Although the force generation mechanism of fin stabilizer at zero speed is different from that of the conventional fin stabilizer, the selection of the feedback control signals can still serve as a reference. Therefore, the roll angle and roll rate are chosen as the feedback signals in the control of the zero-speed fin stabilizer. The range of fin angular velocity is limited within the slash-marked sector as shown in Fig 6 and the following control strategies can be easily obtained:

1. Roll rate based negative feedback control (RRNFC)
2. Roll angle/rate based integrated negative feedback control (RARINFC)

## 4 System modeling and controller design

**Fig 7:**
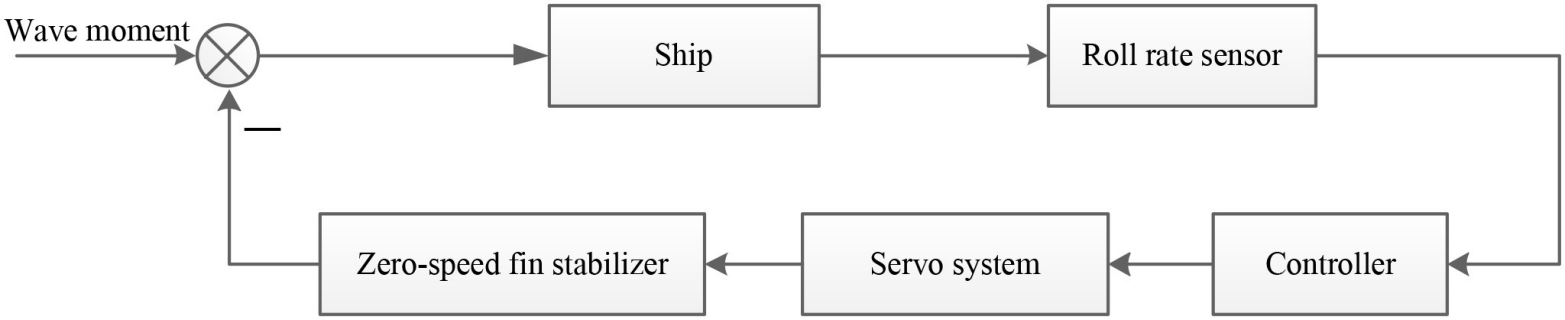
Flowchart of the zero-speed fin stabilizer roll reduction system.

In order to verify the effectiveness of the obtained control strategies in Section 3, the simulation model of zero-speed fin stabilizer roll reduction system was established in MATLAB. The diagram of the established roll reduction system is shown in Fig 7. The roll angle and roll rate are measured by the roll sensor and sent to the controller. The required fin angular velocity to generate the expected force to stabilize the roll motion is calculated in the controller. The corresponding command signal is sent to the electro-hydraulic speed servo system to drive the fins.

### 4.1 System modeling

#### 4.1.1 Mathematical model of ship roll motion

The roll angle of a ship equipped with fin stabilizers is usually less than 10°, and can be described by the linear equation shown in Eq (25). For modeling convenience, Eq (25) can be rewritten as:

$$\begin{array}{c} (I_{x}+\Delta I_{x})\ddot{\phi }+2N_{u}\dot{\phi }+Dh\phi =-Dh\alpha_{1} \end{array}$$ (31)

Where *Dhα*_1_ donates the total external roll moment caused by waves and fin stabilizers, $\alpha_{1}=\alpha_{w}+\alpha_{f}^{\prime }$ is the effective wave slope acting on the ship hull, *α*_*w*_ donates the wave slope and $\alpha_{f}^{\prime }$ donates the fin-induced equivalent wave slope.

Performing the Laplace transformation to Eq (31) under zero initial conditions, the transfer function of the roll motion of the ship can be obtained as [32]:

$$\begin{array}{c} W_{\phi }(s)=\frac{\phi (s)}{\alpha_{1}(s)}=\frac{1}{T_{\phi }^{2}s^{2}+2T_{\phi }n_{u}s+1} \end{array}$$ (32)

Where *T*_*ϕ*_ = *T*_*φ*_/2*π* is the nature rolling period and *n*_*u*_ is the dimensionless roll damping coefficient.

#### 4.1.2 Wave disturbance

Irregular sea waves are usually caused by sea winds. In the study of ship motion, sea waves are usually considered as a homogeneous stochastic process [33]. Waves can be characterized by their Power Spectral Density [34]. In this paper, the ITTC long-crest wave spectrum with double parameters is adopted for the simulation. Its spectral density formula is given as:

$$\begin{array}{c} S_{\zeta }(\omega_{i})=\frac{173h_{1/3}}{T^{4}\omega_{i}^{5}}\exp(-\frac{691}{T^{4}\omega_{i}^{4}}) \end{array}$$ (33)

Where *h*_1/3_ is the significant wave height, *T* is the period the incident wave, *g* is the gravity acceleration, *ω*_*i*_ is the wave frequency of the *i*th regular wave component. The subscript *ζ* means Eq (33) is the wave-height spectrum.

As the waves act on the ship hull in the form of wave slope, therefore, the wave-height spectrum shall be transformed into the wave-slope spectrum. The wave-slope spectrum can be got from the wave-height spectrum through the following formula:

$$\begin{array}{c} S_{\alpha }(\omega_{i})=\frac{\omega_{i}^{4}}{g^{2}}S_{\zeta }(\omega_{i}) \end{array}$$ (34)

Therefore, the wave slope acting on the hull can be calculated as:

$$\begin{array}{c} \alpha (t)=\sum_{i=1}^{60}\sqrt{2S_{\alpha }(\omega_{i})\Delta \omega }\cos(\omega_{i}t+\varepsilon_{i}) \end{array}$$ (35)

Where *ε*_*i*_ is the random phase of the *i*th regular wave. The superscript of the sum operator means that the irregular wave is formed by superposing 60 regular waves with different frequencies and random phases.

#### 4.1.3 Roll rate sensor

The roll rate sensor measures the roll rate and converts it to the corresponding electrical signal. The roll angle can be obtained by integrating the roll rate signal. The transfer function of the roll rate sensor used in this paper is given as [35]:

$$\begin{array}{c} G_{rrs}(s)=\frac{T_{1}s}{s^{2}+T_{2}s+T_{3}} \end{array}$$ (36)

Where *T*_1_ = 400, *T*_2_ = 80 and *T*_3_ = 4000 are the time constants related to the characteristics of the roll rate sensor.

#### 4.1.4 Servo system

The servo system is used to drive the fin stabilizers according to the command signal from the controller. Due to the large moment required to drive the fins, fin stabilizers are usually driven by the electro-hydraulic servo system. Considering the driving power and the required response speed, the pump-controlled hydraulic cylinder based electro-hydraulic servo system is adopted and its transfer function is given as [36]:

$$\begin{array}{c} G_{servo}(s)=\frac{K_{s}}{s(T_{P}s+1(\frac{s^{2}}{\omega_{b}^{2}}+\frac{2\xi_{b}}{\omega_{b}}s+1)} \end{array}$$ (37)

Where *K*_*s*_ = 0.43 is the open-loop gain of the system, *T*_*P*_ = 0.0063 is the time constant, *ω*_*b*_ = 33.4 and *ξ*_*b*_ = 0.3 are the resonant frequency and the damping ratio of the pump-controlled hydraulic cylinder system, respectively.

### 4.2 Controller design

#### 4.2.1 Obtained control strategy

Based on the phase-matching analysis in Section 3, the angular velocity of the zero-speed fin stabilizer is chosen as the manipulated variable to control the fins to reduce the roll motion of the ship at zero speed and the following control strategies are obtained:

**RRNFC**. The roll rate based negative feedback control can be expressed as:

$$\begin{array}{c} G_{C1}(s)=K_{r1}\dot{\phi } \end{array}$$ (38)

Where *K*_*r*1_ > 0 is the proportional gain of controller *G*_*C*1_.

**RARINFC**. The roll angle/rate based integrated negative feedback control can be expressed as:

$$\begin{array}{c} G_{C2}(s)=K_{a2}\phi +K_{r2}\dot{\phi } \end{array}$$ (39)

Where *K*_*a*2_ > 0 and *K*_*r*2_ > 0 are the proportional gains of controller *G*_*C*2_.

#### 4.2.2 Master-slave control

For comparison purpose, a master-slave controller is also designed. As analysis above, the hydrodynamic force generated on the zero-speed fin stabilizer has strong nonlinear relationship with fin angle, angular velocity and acceleration, which can not be piecewise linearized and increases the difficulty of controller design.

Let $x={\left[\phi \dot{\phi }\right]}^{T}$ be the state variable. Rewriting Eq (25) into the state-space representation, we get:

$$\begin{array}{c} \dot{x}=Ax+BK_{C}+CK_{W} \end{array}$$ (40)

Where

$$A=[\begin{array}{cc} 0 & 1\\ -\frac{Dh}{I_{x}+\Delta I_{x}} & -\frac{2N_{u}}{I_{x}+\Delta I_{x}} \end{array}],\;\;B=C=[\begin{array}{c} 0\\ \frac{1}{I_{x}+\Delta I_{x}} \end{array}]$$

Where *K*_*C*_ = 2*l*_*f*_ *F* is the anti-rolling moment, *F* is the anti-rolling force generated on the zero-speed fin stabilizers and *l*_*f*_ is the roll arm of the fins.

It can be seen from Eqs (24) and (40) that the control system of zero-speed fin stabilizer can be equivalent to the series connection of a linear system and a nonlinear system. Therefore, the control process of zero-speed fin stabilizer can be designed in two steps according to the separation strategy: a master controller and a slave controller. In the master controller, the required anti-rolling force (the expected intermediate variable) is estimated online according to the roll motion of the ship. In the slave controller, the required fin angular velocity (the manipulated variable) is obtained based on the expected intermediate variable and Eq (24).

**LQR master controller**. The design target of the master controller is to stabilize the linear system (40) and meet the control requirements. The system matrix *A* and the control input matrix *B* in Eq (40) can be regarded as constant matrices for a particular ship. Therefore, the linear quadratic regulator (LQR) controller is considered. LQR is an optimal control strategy with the quadratic performance indexes and it is widely applied to the active control of deterministic vibratory systems [37]. This is an automatic means of finding an appropriate state-feedback controller that minimizes the performance index [38]. To achieve the optimal roll reduction effect, the linear quadratic performance index is selected as:

$$\begin{array}{c} J=\frac{1}{2}\int_{0}^{\infty }(x^{T}Qx+u^{T}Ru)dt \end{array}$$ (41)

Where *Q* is the semi-positive definite symmetric weight matrix and *R* is the positive definite symmetric matrix. The optimal control law that minimizes the performance index *J* can be calculated as:

$$\begin{array}{c} u=-R^{-1}B^{T}Px=-Kx \end{array}$$ (42)

Where *K* is the optimal control gain and *P* is the symmetric positive definite solution of the Algebraic Riccati Equation (ARE) as follows:

$$\begin{array}{c} PA+A^{T}P-PBR^{-1}B^{T}P+Q=0 \end{array}$$ (43)

**Numeric inversion slave controller**. The expected anti-rolling force can be calculated by the master controller. However, the corresponding expected fin angular velocity cannot be deduced directly by the expected anti-rolling force, as the strong nonlinear relationship between the anti-rolling force generated by the zero-speed fin stabilizers and the fin angle *α*_*f*_, the fin angular velocity *ω*_*f*_ and the fin angular acceleration ${\dot{\omega }}_{f}$. However, it can be seen from the analysis in Sections 2 and 3 that the input nonlinearity of the zero-speed fin stabilizer system is still the univariate nonlinearity. According to the hydrodynamic characteristics of zero-speed fin stabilizer and the consideration in engineering implementation, the fin angular velocity is chosen as the manipulated variable. Therefore, the slave controller is designed to realize the nonlinear inversion from the expected anti-rolling force to the expected fin angular velocity. Let F* be the output of the master controller. Thus, the nonlinear inversion is to solve the nonlinear equation F* − *F*(*ω*_*f*_) = 0. The numerical iterative method is adopted to design the slave controller to realize the nonlinear inversion [23].

Rewriting Eq (23) to the equivalent discrete form, we get:

$$\begin{array}{c} F=K_{1}\omega_{f}(k)|\omega_{f}(k)|+\frac{K_{2}}{T_{s}}(\omega_{f}(k)-\omega_{f}(k-1)) \end{array}$$ (44)

Where *T*_*s*_ is the sampling period.

Let the iterative initial value of the manipulated variable be *ω*_*f*_(*k* − 1) for the sampling point *k*. *f*_1_ and *f*_2_ are defined as:

$$\begin{array}{c} f_{1}=K_{1}\omega_{f}(k)|\omega_{f}(k)|,\;\;f_{2}=\frac{K_{2}}{T_{s}}(\omega_{f}(k)-\omega_{f}(k-1)) \end{array}$$ (45)

Thus, the following inequalities

$$\begin{array}{c} \left\{\begin{array}{c} (\omega_{f}(k)-\omega_{f}^{*}(k))(f_{1}(\omega_{f}(k))-f_{1}(\omega_{f}^{*}(k)))>0\\ (\omega_{f}(k)-\omega_{f}^{*}(k))(f_{2}(\omega_{f}(k))-f_{2}(\omega_{f}^{*}(k)))>0 \end{array},\forall \omega_{f}(k)\neq \omega_{f}^{*}(k)\in \{\omega_{f}\right\} \end{array}$$ (46)

are held true within the set of valid values {*ω*_*f*_}, and the following inequality can be obtained:

$$\begin{array}{c} (\omega_{f}-\omega_{f}^{*})[\left(f_{1}(\omega_{f})+f_{2}(\omega_{f}))-(f_{1}(\omega_{f}^{*})+f_{2}(\omega_{f}^{*})\right)]>0 \end{array}$$ (47)

It can be seen from Eq (47) that the input nonlinearity of the zero-speed fin stabilizer system is bounded and satisfies the local invertibility condition at any sampling point. Considering the iteration scheme and convergence rate, the Newton-Raphson iteration method is adopted and the following equation can be obtained:

$$\begin{array}{c} \omega_{f}(k+1)=\omega_{f}(k)+\frac{1}{F^{\prime }(\omega_{f}(k))}(F^{*}(k)-F(\omega_{f}(k))) \end{array}$$ (48)

## 5 Simulation and experiment

### 5.1 Simulation analysis

**Fig 8:**
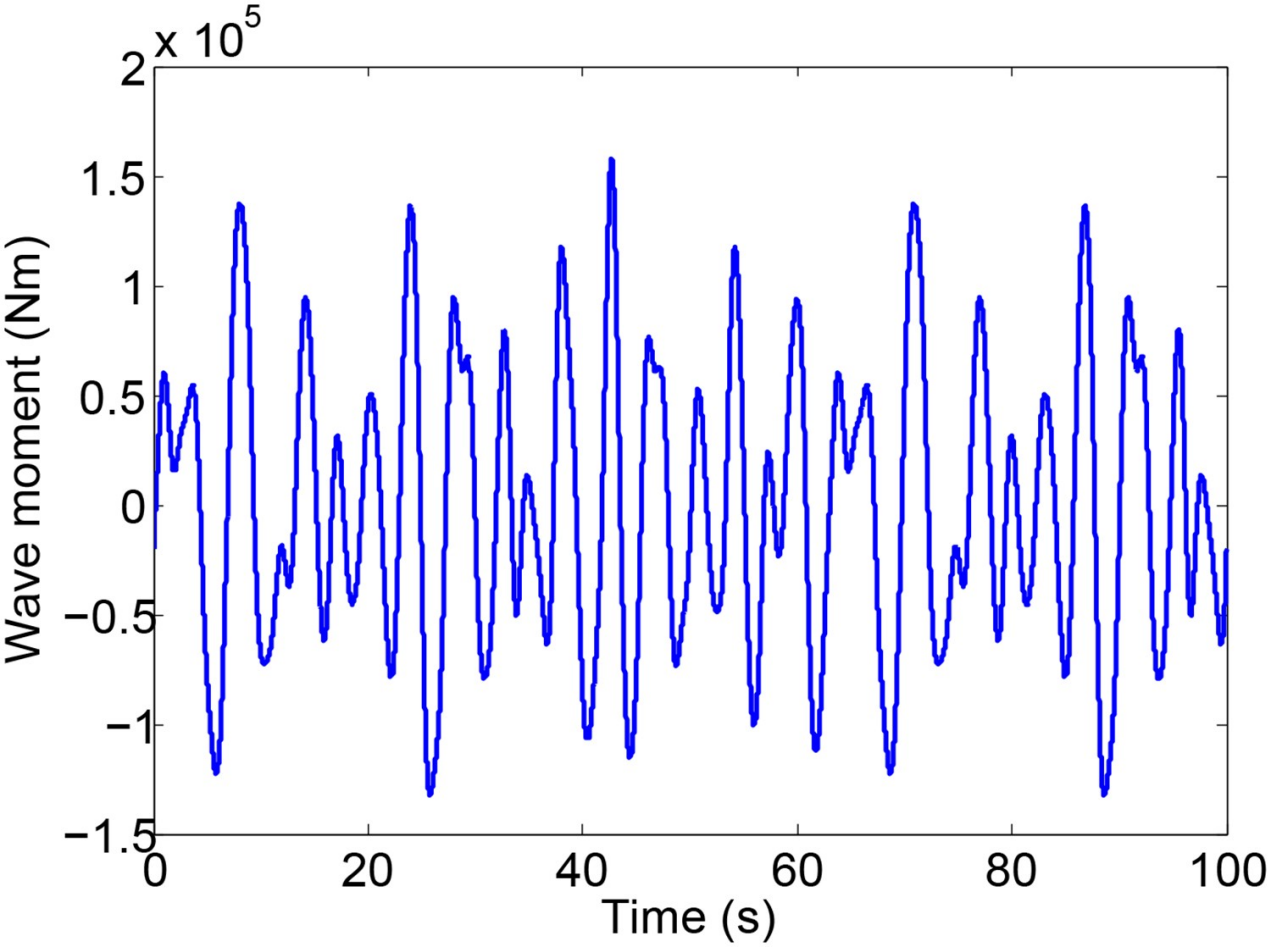
Wave disturbance moment.

**Fig 9:**
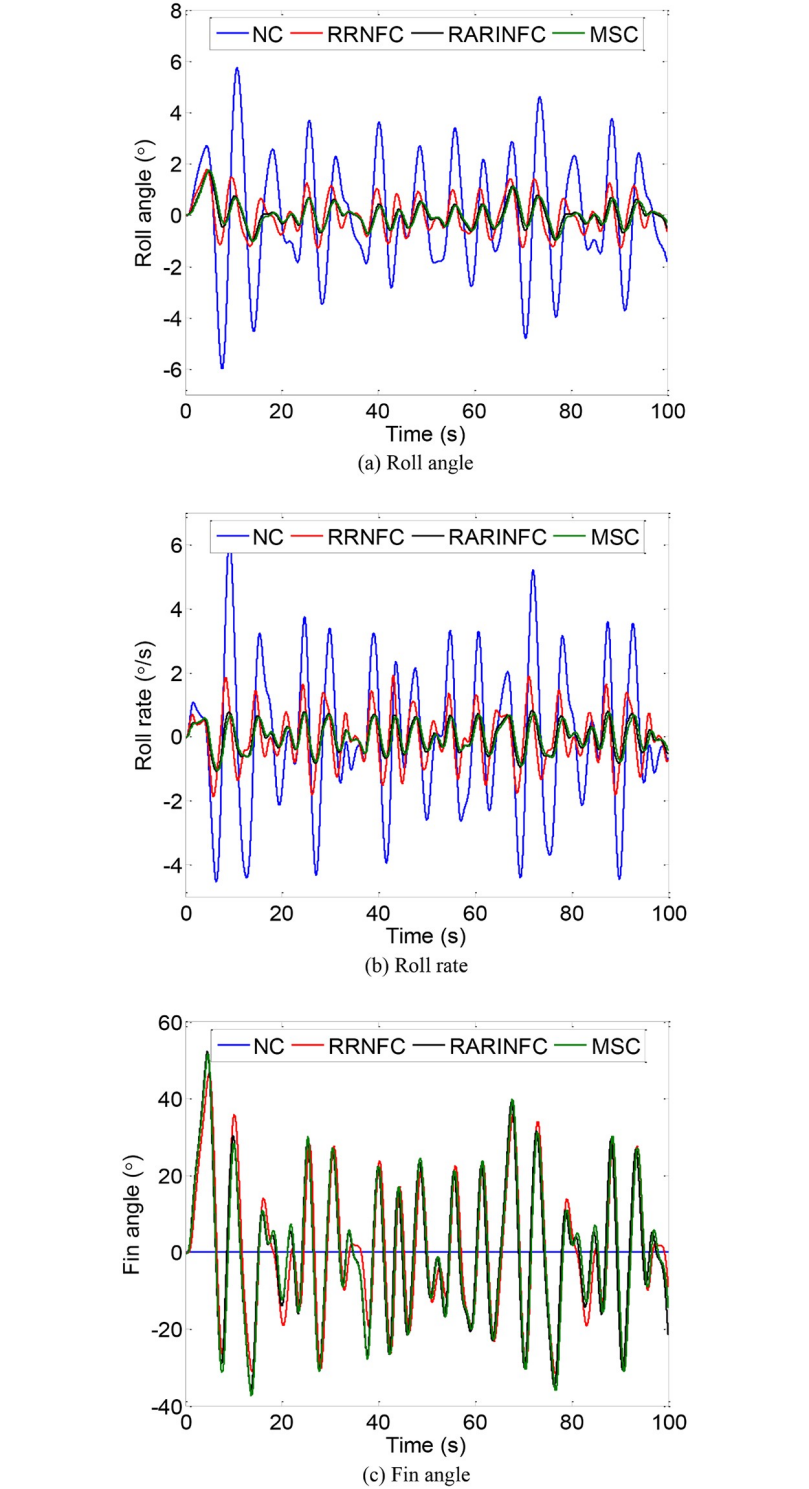
Ship roll motion without and with control. (a) Roll angle. (b) Roll rate. (c) Fin angle.

Simulations are carried out to verify the effectiveness of the obtained control strategies based on the model established in Section 4.1. The main parameters of the ship are listed in Table 1. The significant wave height is 1.5 m, the eigenperiod is 8.5 s, the encounter angle is 90° and the ship speed is 0 kn. The Monte Carlo simulation is adopted to optimize the control parameters of RRNFC and RARINFC. The obtained controller parameters of *G*_*C*1_ and *G*_*C*2_ are given as: *K*_*r*1_ = 2.78, *K*_*a*2_ = 3.26, *K*_*r*2_ = 2.45. The weight matrices of LQR controller are *Q*=diag(10, 1) and *R* = 1. The obtained optimal feedback control gain of LQR controller is K=[0.0416, 0.2706]. Under the action of the wave disturbance shown in Fig 8, the roll motion of the ship with and without roll reduction control is shown in Fig 9. NC, RRNFC, RARINFC and MSC in the legend of Fig 9 represent no control, roll rate based negative feedback control, roll angle/rate based integrated negative feedback control and master-slave control. It can be seen from Fig 9 that the three controllers designed in Section 4.2 can effectively reduce the ship roll motion at zero speed.

In order to quantitatively describe the roll damping performance, the evaluation index suggested by Fossen [39] is adopted:

$$\begin{array}{c} Rollreduction(\%)=\frac{AP-RCS}{AP}\times 100 \end{array}$$ (49)

Where *AP* and *RCS* are the standard deviations of the roll rate before and after the control of roll stabilizer, respectively.

Except for the roll reduction percentage and the roll angle amplitude, the efficiency of the control algorithm is also determined by the action cost of the actuator. To judge the efficiency of the designed controllers, the following cost function is adopted [40].

$$\begin{array}{c} C_{fin}=\sum_{i=1}^{S}\alpha_{i}^{2} \end{array}$$ (50)

Where *S* is the total number of iterations in the time simulation process and *α*_*i*_ is the *i*th fin angle.

According to the above evaluation indexes, the roll damping performance of the zero-speed fin stabilizer under the three designed controllers is obtained, as shown in Table 3. It can be seen from Table 3 that the three controllers are effective in reducing ship roll motion at zero speed. The RARINFC and MSC have better roll reduction effect than the RRNFC. The anti-rolling effect of RARINFC and MSC is around 80%. Although the RRNFC has the worst roll reduction effect among the three controllers, its roll damping performance is still satisfactory, reaching 61.42%. The cost values of the fin deflection under the three controllers calculated from the cost function are also listed in Table 3. These values show that both the RARINFC and MSC have larger fin usage than the RRNFC, but the difference is relatively small. The simulation results show that the control strategies obtained through disturbance and compensation phase-matching analysis are effective.

**Table 3:** Roll damping performance (simulation).

Control method	Roll rate without control (°)	Roll rate with control (°)	Reduction (%)	Fin usage
RRNFC	2.18	0.84	61.42	303.62
RARINFC		0.45	79.46	309.99
MSC		0.42	80.80	315.87

For comparison and verification purpose, the roll angles before and after the control of roll stabilizers under the above three controllers, variant constraint model predictive control (VCMPC), neural network control (NNC) and fuzzy sliding mode control (FSMC) are also given, as shown in Table 4. It should be noted that the target vessel in [23] and [24] is a 52-meter-long small ship, and that is why the roll response without control in the same sea conditions is larger. It can be seen from the comparison that the anti-rolling effect of the zero-speed fin stabilizer under wave disturbance with the significant wave height of 1.5 m is about 70%∼80%., which also demonstrates the effectiveness of the controllers designed in this paper.

**Table 4:** Performance of zero-speed fin stabilizer.

Roll angle without control (°)	Control method	Roll angle with control (°)	Anti-rolling effect (%)
4.45	RRNFC	1.43	67.73
	RARINFC	0.98	77.81
	MSC	0.93	79.05
4.80	FSMC	1.14	76.17
5.48	VCMPC	1.56	71.51
5.23	NNC	1.42	72.76

### 5.2 Water tank test

**Fig 10:**
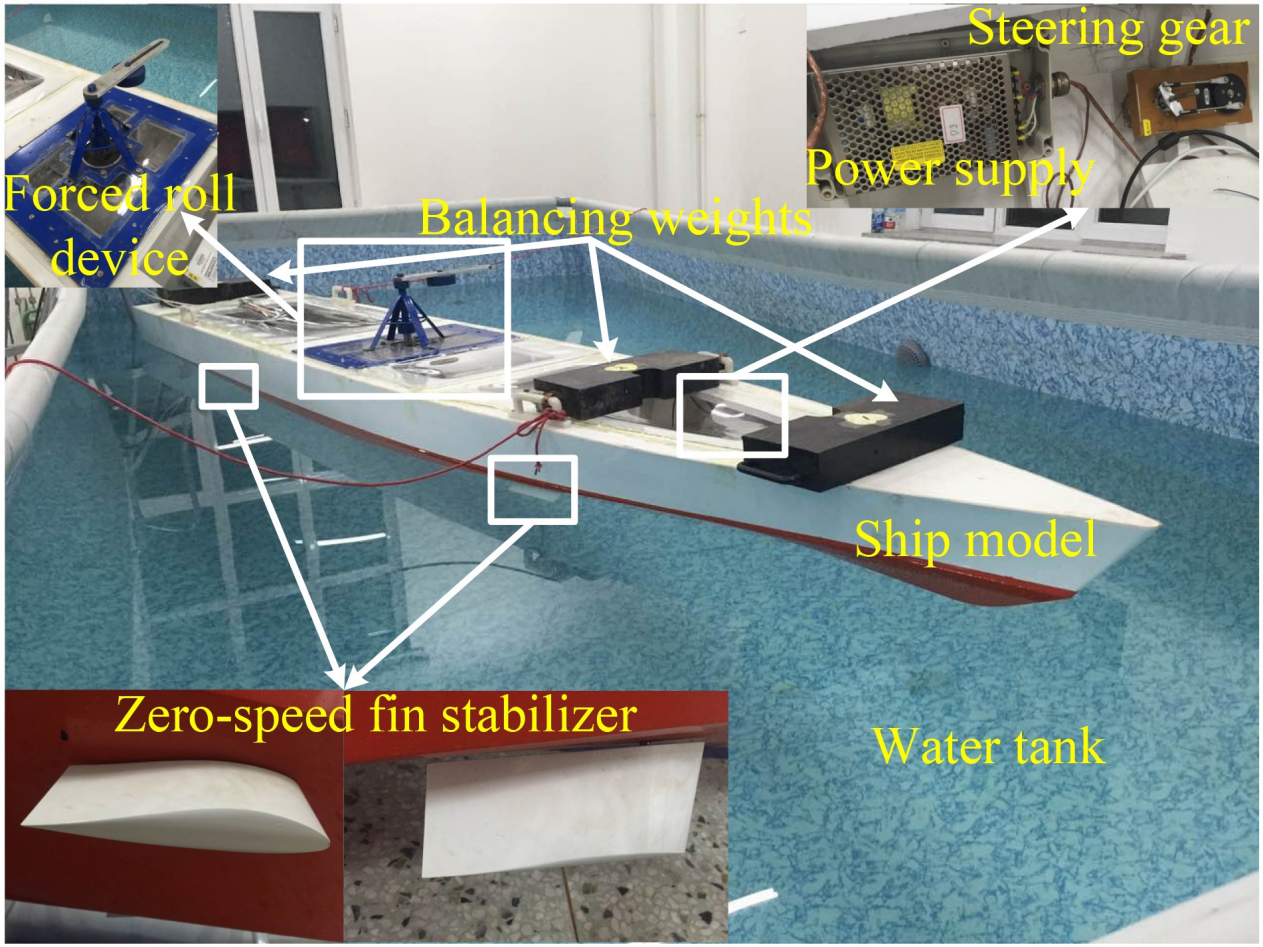
Zero-speed roll reduction system.

To further verify the applicability and effectiveness of the obtained control strategies in practical applications, the model tank tests were carried out. The zero-speed roll reduction system is shown in Fig 10. The system consists of a water tank, a scaled ship model, a forced roll device, four balancing weights, two pairs of scaled zero-speed fin stabilizers and the corresponding driving units, a roll rate sensor, a data acquisition unit, and two computers (one for controlling the forced roll device and the other for data acquisition and fin control).

**Fig 11:**
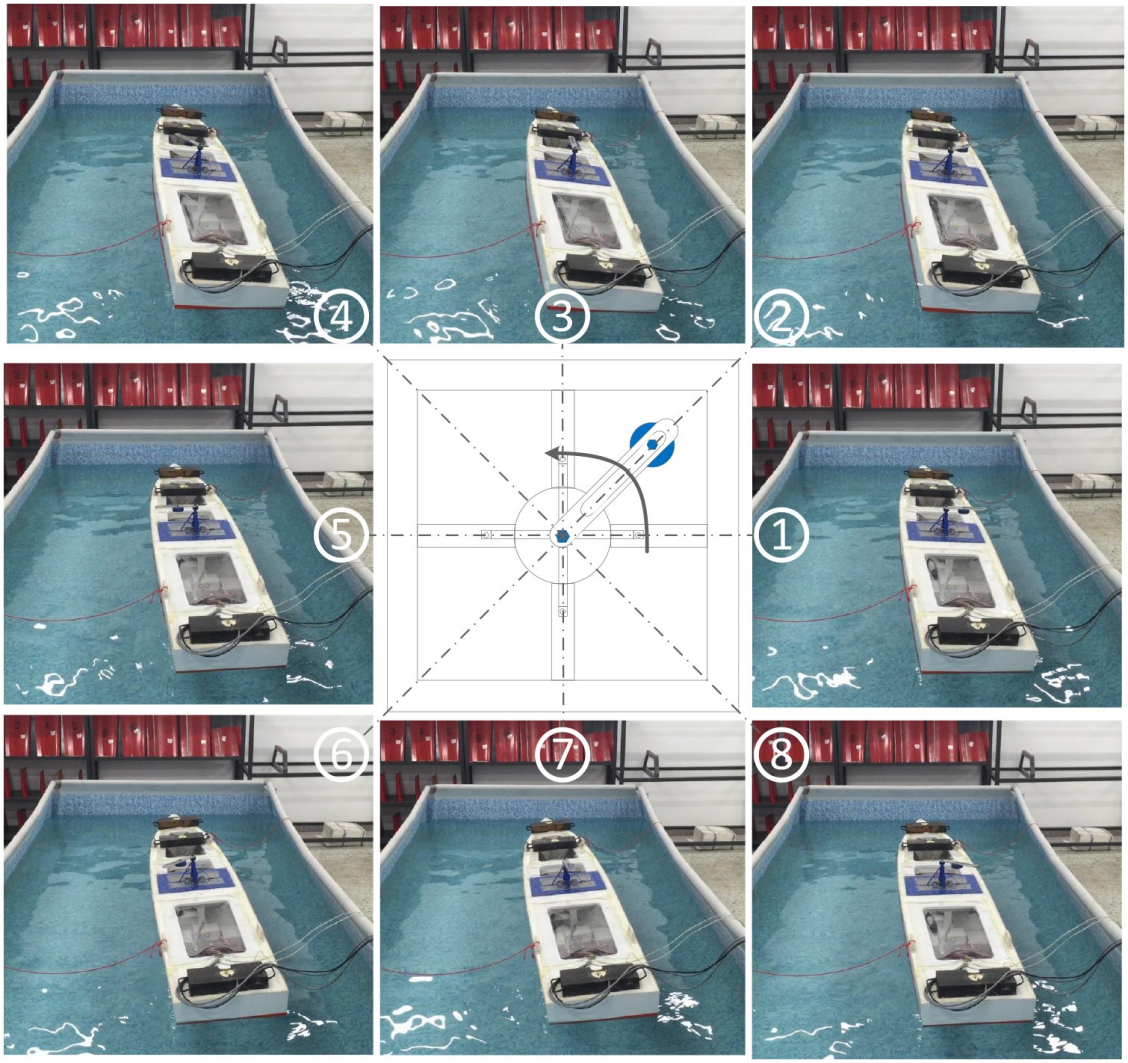
Motion of the forced roll device.

The scaled ship model rolls periodically under the action of the forced roll device. The motion of the forced roll device is shown in Fig 11. The data acquisition unit collects the roll angle and roll rate signals measured by the roll rate sensor mounted in the center of the ship model. The controller calculates the required fin angular velocity according to the preset control algorithm using the obtained roll information. The scaled zero-speed fin stabilizers are driven by the driving units according to the command from the controller to stabilize the roll motion of the ship model.

**Fig 12:**
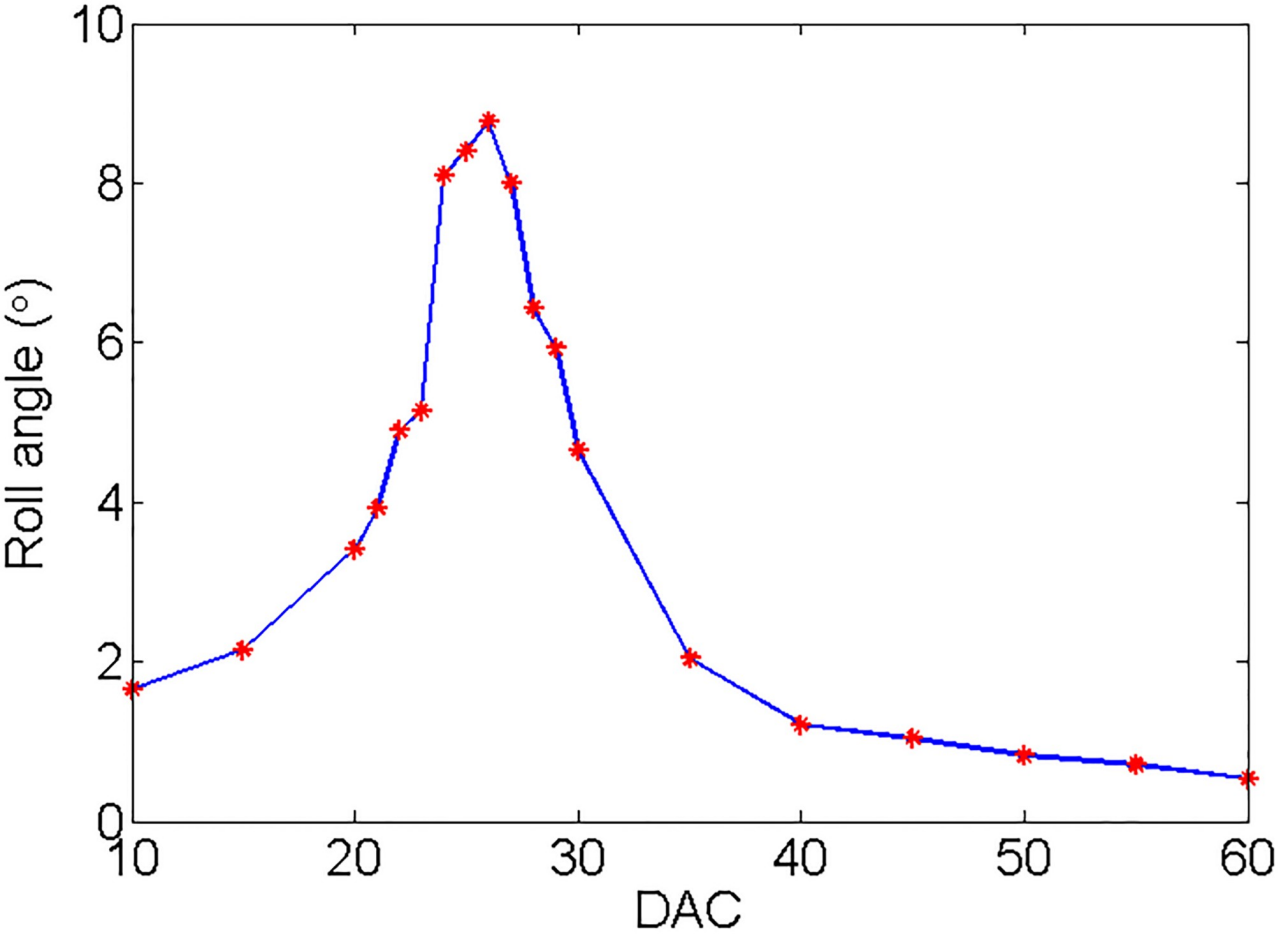
Results of the forced roll test.

**Fig 13:**
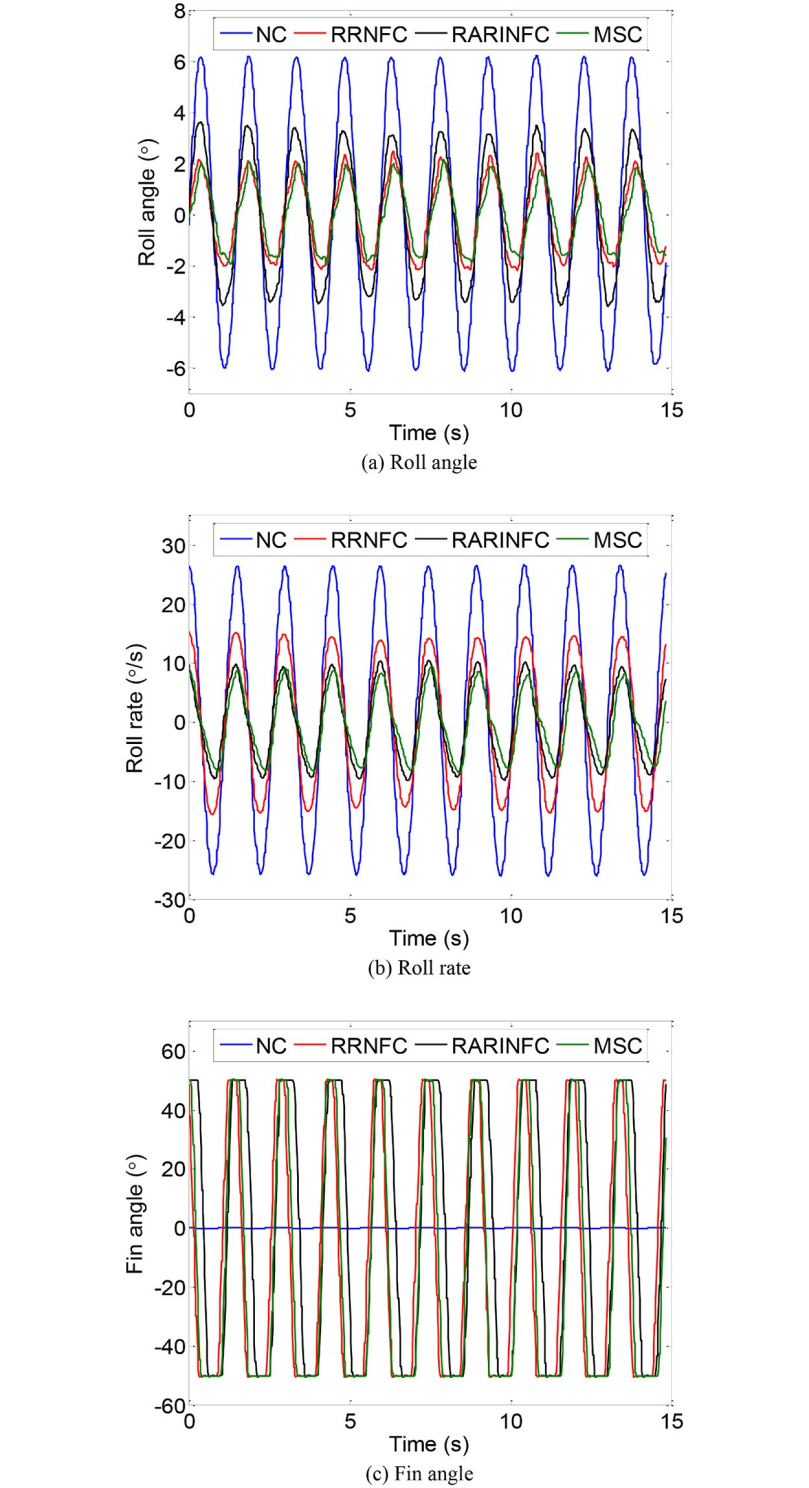
Roll motion of the scaled ship model without and with control. (a) Roll angle. (b) Roll rate. (c) Fin angle.

First, the free decay test was conducted to determine and verify the nature rolling period and damping of the scaled ship model. The obtained nature roll period and dimensionless roll damping are 1.6998 s and 0.121, which is consistent with theoretical calculations. Then, the forced roll test under the action of forced roll device was conducted. The results of the forced roll test are shown in Fig 12. It can be seen from Fig 12 that the scaled ship model has the largest roll response when the driving signal DAC of the forced roll device is 26. For comparison purpose, all the roll reduction tank tests were performed under this condition. Fig 13 shows the roll response of the scaled ship model under the above condition without and with roll reduction control. The corresponding performance of the three controllers is obtained according to the evaluation indexes adopted in Section 5.1, and the results are shown in Table 5. It should be noted that the scaled ship model slightly tilts to the starboard in calm water condition due to the imbalance of counterweight, which accounts for the asymmetry of the fin angle signal in Fig 13.

**Table 5:** Roll damping performance (experiment).

Control method	Roll rate without control (°)	Roll rate with control (°)	Reduction (%)	Fin usage
RRNFC	18.83	10.91	41.50	1669.20
RARINFC		6.48	64.69	1737.98
MSC		5.64	69.75	1867.17

It can be seen from Fig 13 and Table 5 that the results of roll reduction tank tests under the three controllers are lower than the simulation results. This may be caused by the simplification in the modeling process and other unconsidered factors in the simulations. The trend in the cost of fin deflection is consistent with the simulation. Therefore, it can be concluded that the tank test results are generally consistent with the simulation results. Among the three control methods, the MSC with a LQR master controller and a numeric inversion slave controller has the best anti-rolling effect. The roll reduction effect of the RRNFC is relatively low due to its poor phase matching ability. While the anti-rolling effect of the RARINFC has been improved by adjusting the two control gains to match the phase relationship analyzed in Section 3. Therefore, the results of simulations and tank tests demonstrate that the control strategies obtained in this paper are effective and practical, and can be a reference for engineering practice.

## 6 Conclusion

1. The hydrodynamic force model of zero-speed fin stabilizer was established using NACA0015 fin as the prototype. A pair of zero-speed fin stabilizers with aspect ratio of 0.5 was designed for an 84-meter-long fishery ship, and the simplified hydrodynamic model of the designed zero-speed fin stabilizer is obtained by fitting the data from CFD simulations.
2. The control strategy of the zero-speed fin stabilizer was obtained based on disturbance and compensation, which is accomplished by analyzing the phase relationship between the roll motion caused by the wave disturbance and the fin’s movement that produces the compensation torque. According to the hydrodynamic characteristics of the zero-speed fin stabilizer and the consideration for engineering implementation, the fin angular velocity is selected as the manipulated variable. Based on the results of phase matching analysis, the roll rate based negative feedback control (RRNFC) and the roll angle/rate based integrated negative feedback control (RARINFC) were obtained. A master-slave controller (MSC) was also inspired and designed for comparison purpose.
3. The roll reduction system using zero-speed fin stabilizers was established in MATLAB to verify the effectiveness of the obtained control strategies. Simulation results show that all the three controllers can effectively reduce the roll motion of the ship at zero speed. RARINFC and MSC have better roll reduction performance, and both of them have a roll damping effect of around 80%. The anti-rolling effect of the RRNFC also exceeds 60%.
4. Water tank tests were carried out to further verify the practicability of the designed controllers. Although the roll reduction performance of the tank tests is lower than that of the simulations, the tank test results are generally consistent with the simulation results. Therefore, the control strategies obtained based on disturbance and compensation are proved to be effective and practical, and can be a reference for the controller design of zero-speed fin stabilizer in practical applications.
5. Limited by experimental conditions, the scaled ship model under the action of the forced roll device can only roll periodically. However, the unpredicted and high frequency roll motions are the biggest challenges to design roll damping stabilizers. Therefore, the established zero-speed roll reduction system can only be used to preliminary verify the effectiveness of the designed control algorithm under laboratory conditions. In the future, the scaled ship model test in the natural environment will be carried out to simulate the actual operating conditions of the fin stabilizers. If conditions permitting, the full scale test will also be conducted to further verify the effectiveness and practicability of the designed controllers.
